# Tuning Surface Reactivity and Electric Field Strength via Intermetallic Alloying

**DOI:** 10.1021/acsenergylett.3c01639

**Published:** 2023-09-27

**Authors:** Ezra L. Clark, Rasmus Nielsen, Jakob Ejler Sørensen, Julius Lucas Needham, Brian Seger, Ib Chorkendorff

**Affiliations:** † Surface Physics and Catalysis, Department of Physics, Technical University of Denmark, 2800 Kongens Lyngby, Denmark

## Abstract

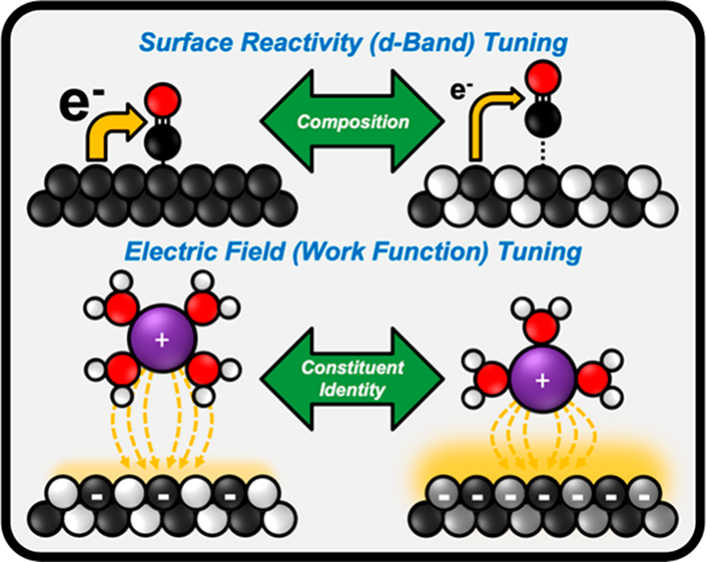

Many electrosynthesis reactions, such as CO_2_ reduction to multicarbon products, involve the formation of dipolar and polarizable transition states during the rate-determining step. Systematic and independent control over surface reactivity and electric field strength would accelerate the discovery of highly active electrocatalysts for these reactions by providing a means of reducing the transition state energy through field stabilization. Herein, we demonstrate that intermetallic alloying enables independent and systematic control over d-band energetics and work function through the variation of alloy composition and oxophilic constituent identity, respectively. We identify several intermetallic phases exhibiting properties that should collectively yield higher intrinsic activity for CO reduction compared to conventional Cu-based electrocatalysts. However, we also highlight the propensity of these alloys to segregate in air as a significant roadblock to investigating their electrocatalytic activity.

Electrocatalysis enables chemical transformations to be directly driven by renewable electricity, providing a green alternative to conventional chemical synthesis methodologies.^1−3^ The large majority of chemical transformations relevant to modern society involve the formation of dipolar transition states, the energetics of which ultimately dictate the energy efficiency of the process. Charged electrode surfaces produce exceptionally strong local electric fields (∼1 V/Å) that can significantly stabilize these dipolar transition states, improving the free energy landscape of the corresponding chemical transformation.^4,5^ Electrochemical CO_2_ reduction is an example of an electrocatalytic reaction that involves the formation of a dipolar transition state during the rate-determining step.^4−6^ The reaction has garnered significant contemporary interest because it enables carbon-neutral alcohols, aldehydes, alkenes, and carboxylic acids to be produced directly from renewable electricity.^7,8^ However, metallic copper is the only electrocatalyst capable of catalyzing the reaction, and it suffers from an excessive overpotential requirement.^7−12^ CO_2_ reduction over Cu proceeds through the formation of a reversibly adsorbed CO* intermediate.^7,13−25^ The rate of CO reduction to multicarbon products over Cu depends only on the absolute applied potential (V vs SHE) and is independent of proton chemical potential (pH).^17,23,26^ This observation suggests that the rate-determining step involves only electron transfer. This rate-determining step is hypothesized to be the dimerization of two adjacently adsorbed CO* intermediates to form a highly dipolar and polarizable OCCO* intermediate.^25,27−34^ Enhancing the stability of the OCCO* intermediate relative to the CO* reactants would yield superior intrinsic electrocatalytic activity and energy efficiency. However, a linear scaling relationship exists between OCCO* and CO* because both species bind to the electrode surface through carbon atoms. Methods for breaking this scaling relationship must be identified so that electrocatalysts with superior intrinsic activity can be developed.

The rate of multicarbon product formation observed during CO_2_ reduction is significantly influenced by the identity of the electrolyte cation.^5,35−39^ In fact, the ethene partial current density increases by roughly an order of magnitude at a fixed applied potential as the identity of the electrolyte cation moves down the alkali metal group. This cation sensitivity is hypothesized to be the result of the dependence of the local electric field strength on cation identity.^39^ Larger alkali cations exhibit lower solvation energies, which enables them to more easily shed their solvation shell and closely approach the negatively charged cathode surface, elevating the local electric field strength. An elevated electric field enhances multicarbon product formation because OCCO* exhibits greater electric field sensitivity than CO*.^5,39^ Thus, local electric fields enable the scaling relationship that exists between OCCO* and CO* to be broken. The extent of cation promotion also depends on the surface atomic structure of the electrocatalyst itself, with more densely packed surfaces exhibiting greater cation sensitivity.^39^ The surface structure dependence of cation promotion has been explained in terms of the potential of zero charge of the electrocatalyst, which systematically shifts to more anodic potentials as the work function of the surface increases.^40^ While cation identity and the potential of zero charge collectively determine the local electric field strength, the impact of the potential of zero charge is much more significant.^39^ Thus, electrocatalysts with a similar CO* binding energy as Cu but a higher work function should exhibit significantly superior intrinsic kinetics for CO reduction to multicarbon products. The identification of such materials would be accelerated by a material platform enabling independent and systematic tuning of the surface reactivity and work function. If identified, this material platform could be applied to a wide array of organic electrosynthesis reactions involving the formation of a dipolar and polarizable transition state during the rate-determining step, facilitating the development of sustainable electrocatalytic technologies for commodity and fine chemical synthesis.

An intermetallic is an alloy that exhibits an ordered solid-state structure. This ordered structure arises due to the electronic dissimilarity of the constituent metals, which results in the formation of a strong heteronuclear bond between them. This strong heteronuclear bond significantly modifies the electronic properties of the constituent metals through a ligand effect.^41−49^ Thus, intermetallics can be thought to consist of a catalytically active late transition metal and an electronic modifier, which can be either an early or post-transition metal. The electronic modifications exhibited by the late transition metal are characterized by a systematic shift of the d-band away from the Fermi level toward higher binding energies.^44,46−48^ The extent of these electronic modifications is a function of both the strength (alloy formation energy) and the number (alloy composition) of intermetallic bonds the late transition metal participates in. Transition metal surface reactivity is largely determined by d-band energetics.^49−51^ Thus, the ability to systematically modify the energetics of the d-band provides a direct method for systematically tuning the surface reactivity to the optimum value for a given catalytic application. As a result, it should not be surprising that intermetallic alloys exhibit desirable activity and selectivity for a variety of thermally catalyzed reactions. In fact, several Ni-, Pd-, and Pt-based intermetallic alloys exhibit Cu-like reactivity for methanol steam reforming,^52−57^ a reaction that involves similar reaction intermediates as electrochemical CO_2_ reduction. This Cu-like catalytic activity has been attributed to the electronic modifications arising from intermetallic bonding, which yield surfaces with Cu-like d-band energetics.^55,57^

While the d-band energetics of intermetallics depend on the strength of the intermetallic bond and alloy composition, the work function of the intermetallic surface is expected to be a linear combination of the work functions of the constituent metals. Many intermetallic systems exhibit similar formation energies.^58^ As a result, the d-band energetics of these alloys are expected to be similar at commensurate compositions. However, the various possible early and post-transition metal constituents exhibit work functions spanning a range of roughly 2 eV.^59,60^ Thus, the identity of the post-transition metal constituent is expected to play a significant role in determining the work function of the resulting intermetallic surface. Therefore, intermetallic alloys are a promising material platform that could enable the independent and systematic tuning of surface reactivity (d-band energetics) and electric field strength (work function). If validated, this capability could be leveraged to develop superior electrocatalysts for CO reduction through the selective field stabilization of OCCO* relative to CO*. To this end, an ideal candidate electrocatalyst should exhibit d-band energetics similar to those of Cu but an elevated work function.

Hundreds of different bimetallic intermetallic alloys exist, each with multiple stable phases. Experimentally exploring this entire phase space is a daunting task. However, promising intermetallic systems can be quickly identified by using a series of selection criteria. First, both constituent metals must be in a metallic state before the onset potential of electrochemical CO reduction over Cu, which is roughly −1.2 V vs SHE in 0.1 M KOH.^23^ If this criterion is not met, then an intermetallic bond will not exist between the constituent metals until more cathodic potentials are applied, negating the possibility of discovering an electrocatalyst superior to Cu. Second, the intermetallic alloy formation energy should be more exergonic than the adsorption energy of CO to Cu. The unique ability of Cu to catalyze electrochemical CO reduction is hypothesized to be due in part to its uniquely moderate CO adsorption energy.^11,15,17,61^ Thus, intermetallic phases exhibiting similar CO adsorption energies as Cu have the best chance of catalyzing CO reduction. However, if the cohesive energy of the candidate intermetallic phase is not greater than this CO adsorption energy, then the intermetallic alloy will spontaneously segregate upon CO adsorption. Finally, the intermetallic surface should exhibit a work function greater than that of Cu, assuming that the work function is equivalent to a linear combination of the work functions of the constituent metals. The higher the work function, the stronger the local electric fields will be at a fixed applied potential, the more stable OCCO* will be relative to CO*, and the higher the intrinsic CO reduction activity is expected to be. Roughly 300 bimetallic intermetallic systems consisting of a group 8, 9, 10, or 11 element and a group 3, 4, 5, 6, 12, 13, 14, or 15 element were evaluated using these selection criteria (Supporting Information (SI), section SI-1). Roughly 50% of these intermetallic systems were removed from the candidate list because many early transition metals will not be in a metallic state until potentials more negative than −1.2 V vs SHE are applied. Roughly 90% of the remaining candidates were eliminated because many form intermetallic bonds of insufficient strength to prevent alloy segregation upon CO adsorption. Only 20 Rh-, Ir-, Pd-, and Pt-based intermetallic systems satisfy all of these requirements. Of these, Pd-based intermetallic alloys with Ge, Sn, and In were selected for further investigation because the strength of their intermetallic bonds vary by only 30 mV while the work functions of the post-transition metal constituents span a range of 1 eV.

Intermetallic thin films were synthesized by cosputtering (SI, section SI-2). Their bulk compositions were measured using energy dispersive spectroscopy (SI, section SI-3), and their crystal structures were measured using grazing incidence X-ray diffraction (SI, section SI-4). These measurements confirmed the phase purity of all of the intermetallic phases investigated herein (SI, section SI-5). Scherrer and Williamson–Hall analyses of the diffraction data indicated that the average crystallite size decreased and that the maximum lattice strain increased as the Pd content of the thin films increased (SI, section SI-6). However, these systematic variations were relatively small, corresponding to average size and maximum stress variations of only 15 nm and 0.2%, respectively, across the entire composition range investigated. The intermetallic thin films were then transferred into an ultrahigh vacuum system where electronic structure characterization was performed. Surface cleaning was performed before electronic structure characterization using a rastered Ar ion beam. Subsequent X-ray photoelectron spectroscopy measurements confirmed that the near-surface compositions of these films were equivalent to the bulk after this cleaning step (SI, section SI-7). Furthermore, low energy He ion scattering indicated that the surface composition of these thin films is approximately equivalent to that of the bulk after this cleaning step (SI, section SI-8). The influence of intermetallic alloy composition on the d-band energetics of Pd was measured for the Pd_*x*_Sn_1–*x*_ system because it exhibits the greatest number of stable intermetallic phases. The d-band energetics were found to systematically shift away from the Fermi level and narrow as the Sn content of the intermetallic alloy increased, as shown in Figure 1A. In fact, the Pd d-band center increases by roughly 2 eV by varying the intermetallic alloy composition throughout the accessible composition range. While many of the analyzed phases exhibit d-band energetics resembling Cu, PdSn and PdSn_2_ provide the best match. The systematic tuning of the Pd d-band should yield systematic changes in the CO adsorption energy of Pd. To validate this notion, CO temperature-programmed desorption was performed, as shown in Figure 1B. The integral of the CO desorption curve was directly proportional to the Pd content of the intermetallic alloy, corroborating the notion that CO only adsorbs to surface Pd sites. The average CO desorption temperature was found to systematically shift to lower temperatures as the Sn content of the intermetallic alloy increased. The average CO desorption temperatures observed over PdSn and PdSn_2_ (180 and 150 K, respectively) were significantly lower than those observed over pure Pd (350 K) and in close agreement with pure Cu (160 K). Thus, the electronic modifications induced by intermetallic bonding significantly reduce the surface reactivity of Pd, making it nearly identical to Cu.

**Figure 1:**
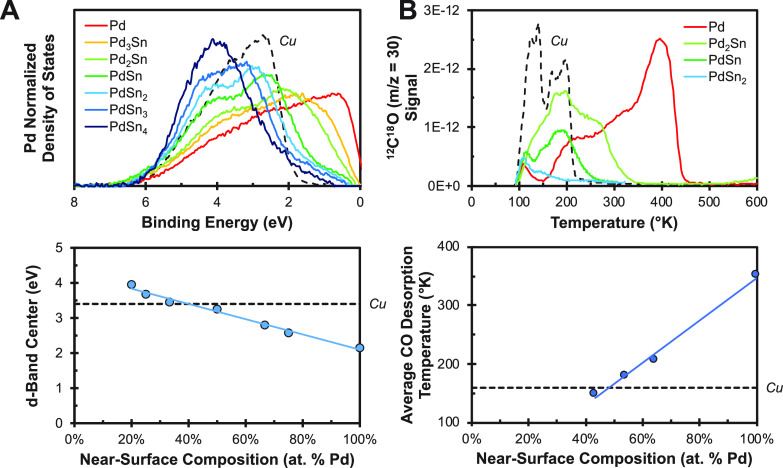
(A) Systematic tuning of the Pd d-band with the composition of Pd_*x*_Sn_1–*x*_. (B) CO temperature-programmed desorption over Pd_*x*_Sn_1–*x*_. Cu is shown for comparison.

As previously mentioned, Pd forms intermetallic bonds of similar strength to Ge, Sn, and In. Thus, the Pd d-band energetics of these intermetallics are expected to be independent of the identity of the post-transition metal for commensurate compositions. Pd-based intermetallic phases of the form Pd_3_M, Pd_2_M, and PdM (where M = Ge, Sn, and In) are all stable. However, phases of the form PdM were selected for further investigation because they are expected to exhibit the desired d-band energetics. This is exactly what is observed, as shown in Figure 2A. However, the Pd d-band does not shift as significantly when alloyed with In, likely due to either the different crystal structure of this intermetallic phase (cubic vs orthorhombic) or the different valence electron configuration of this post-transition metal (3d^10^4s^2^4p^1^ vs 3d^10^4s^2^4p^2^). Conversely, the work function of these intermetallics is significantly influenced by the identity of the post-transition metal, as shown in Figure 2B (SI, section SI-9). Several of these Pd-based intermetallic alloys exhibit Cu-like d-band energetics but significantly higher work functions, such as PdGe (+0.26 eV) and PdSn (+0.12 eV). However, PdGe is the most promising phase because it exhibits a d-band center only 0.1 eV closer to the Fermi level than Cu and a work function 0.26 eV higher. These properties will yield a CO* adsorption energy similar to Cu but an elevated electric field strength under cathodic polarization. Intermetallic alloying can also influence surface reactivity by changing the adsorption site nuclearity. CO adsorbs to monometallic Pd at the bridge sites. However, CO adsorbs to PdZn surface alloys at Pd atop sites, despite the fact that such alloys still expose an abundance of Pd–Pd bridge sites.^62^ These observations suggest that the electronic modifications arising from intermetallic bonding play a larger role in modulating the CO adsorption energy in comparison to the modifications to adsorption site nuclearity. However, the adsorption site nuclearity will play a critical role in determining the reaction selectivity. Promising intermetallic electrocatalysts must expose neighboring CO adsorption sites in order to catalyze CO dimerization. Luckily, a surface termination of the bulk PdGe structure is expected to expose alternating rows of each constituent metal,^63,64^ providing an abundance of neighboring CO adsorption sites for efficient C–C coupling.

**Figure 2:**
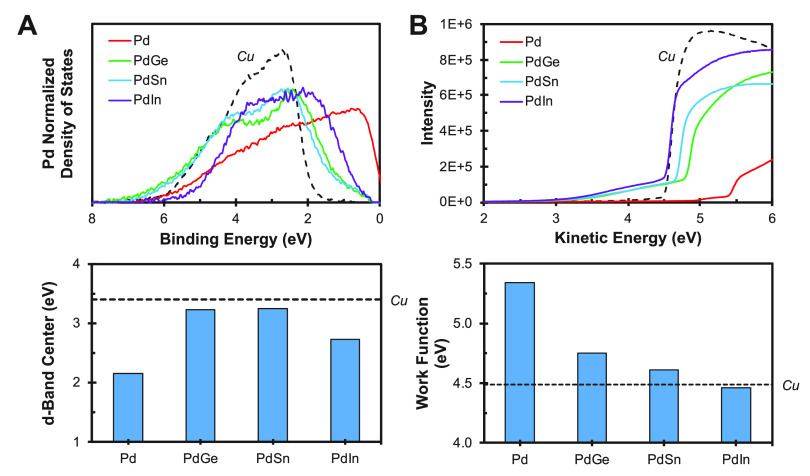
(A) Independence of the Pd d-band with post-transition metal constituent identity in PdM (M = Ge, Sn, and In) intermetallics. (B) Systematic tuning of work function with post-transition metal identity in PdM (M = Ge, Sn, and In) intermetallics. Cu is shown for comparison.

Intermetallic materials have received significant interest for thermal catalytic applications in recent years. These studies have demonstrated that the activity and selectivity of these materials is significantly influenced by the catalyst pretreatment methodology.^52−54^ Catalyst pretreatment plays a significant role in modulating the activity and selectivity of these materials due to their susceptibility to segregation upon air exposure. Surface segregation in air occurs due to the oxophilicity differences of the constituent metals, which are greater than the strength of the intermetallic bond for all intermetallic systems. This means that all intermetallic alloys are susceptible to segregation in air, as shown in Figure 3. To demonstrate this segregation issue, PdGe thin films were briefly exposed to air (∼1 min) and subsequently analyzed with a combination of X-ray photoelectron spectroscopy and low energy He ion scattering. XPS measurements clearly indicate that significant Ge oxidation and surface segregation occur upon air exposure, as shown in Figure 4A. In fact, the segregation is so severe that it is entirely unclear if any Pd is present at the surface after air exposure. Low energy He ion scattering depth profiles clearly demonstrate that air exposure results in the formation of a conformal GeO_2_ overlayer that completely buries the electronically modified Pd centers beneath the surface, as shown in Figure 4B (SI, section SI-10). Thus, it should be no surprise that these intermetallics do not exhibit CO reduction activity after air exposure (SI, section SI-11).

**Figure 3:**
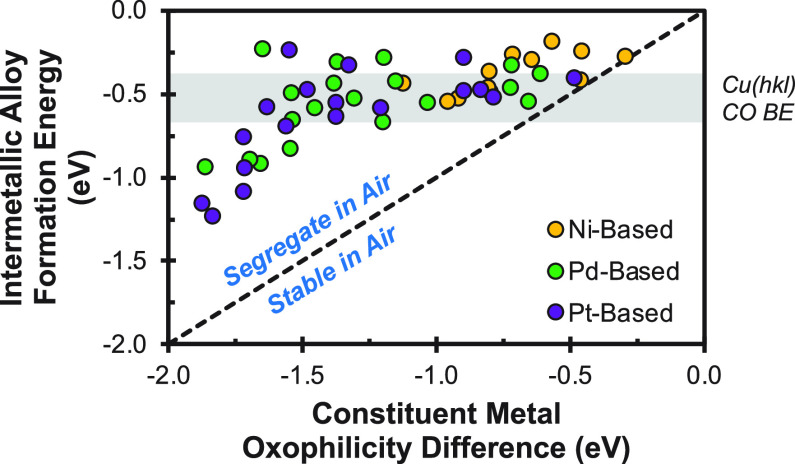
Comparison of the alloy formation energy and constituent metal oxophilicity difference of various Ni-, Pd-, and Pt-based intermetallic alloys. Note that in all cases there is a driving force for segregation upon air exposure.

**Figure 4:**
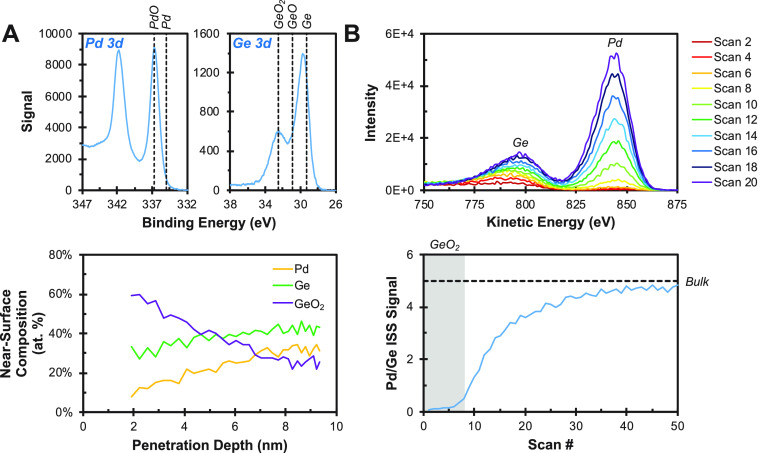
(A) Angle-resolved XPS measurements of an air exposed PdGe thin film. Pd and Ge 3d regions demonstrate that Ge is present in multiple oxidation states and that the oxidized Ge accounts for a greater fraction of the metal content as the penetration depth is reduced. (B) Low energy He ion scattering depth profile of an air exposed PdGe thin film.

High temperature catalyst pretreatment in a reducing environment is typically performed to reduce conformal surface oxides and provide the atomic mobility required to restore the intermetallic surface. While thermal pretreatment is ubiquitous in thermal catalysis, the same is not true for electrocatalysis. In fact, electrocatalyst pretreatment often only entails cycling the applied potential until a stable voltammogram is obtained. While this electrochemical pretreatment will certainly reduce these surface oxides, it will not provide the thermal energy required to restore the intermetallic surface and can also result in significant leeching of the more oxophillic constituent metal into the electrolyte (SI, section SI-12). Cycling of PdGe beyond the oxidation potential of Ge results in the near-instantaneous dissolution of all of the Ge in the sample. Thermal pretreatments without subsequent air exposure are difficult and are rarely performed on electrocatalysts. In fact, such pretreatments have not been performed in any peer-reviewed investigation of intermetallic electrocatalysts to date. Therefore, the surface structures of the intermetallic electrocatalysts in the few studies that have been performed are ill-defined and likely do not exhibit the unique electronic properties that make these materials promising for electrocatalytic applications. There is a significant need to develop experimental methods that enable the electrocatalytic activity of these materials to be easily and accurately assessed, so that the utility of their unique electronic properties for electrocatalytic applications can be confirmed and rigorously investigated.

In conclusion, intermetallic alloys are a promising material platform that enables independent and systematic tuning of surface reactivity (d-band) and electric field strength (work function) through variation of the alloy composition and oxophilic constituent identity, respectively. This capability can be leveraged to develop superior electrocatalysts for reactions involving the formation of dipolar transition states during the rate-determining step, such as the reduction of CO_2_ to multicarbon products. However, the electronic dissimilarity of the constituent metals also makes these alloys incredibly susceptible to segregation in air. As a result, new experimental methods are required to accurately investigate the electrocatalytic activity of these materials.

## Supporting Information Available

Experimental methods, intermetallic alloy selection, energy dispersive spectroscopy of intermetallic thin films, grazing incidence X-ray diffraction of intermetallic thin films, intermetallic thin film phase identification, Scherrer and Williamson–Hall analysis of XRD data, extended X-ray photoelectron spectroscopy of Ar sputtered intermetallic thin films, low energy He ion scattering of Ar sputtered intermetallic thin films, validation of work function measurements, low energy He ion scattering depth profile of air exposed PdGe, CO reduction over air exposed and segregated PdSn, deleterious impact of electrochemical pretreatment on PdGe (PDF)
