# Supplementary material for: Tuning Surface Reactivity and Electric Field Strength via Intermetallic Alloying
Source: ACS Energy Lett. 2023 Sep 27;8(10):4414–20. doi: 10.1021/acsenergylett.3c01639 (PMC10580307; doi:10.1021/acsenergylett.3c01639)
Supplement: Supplementary file 1 — nz3c01639_si_001.pdf [file nz3c01639_si_001.pdf]

SUPPLEMENTARY INFORMATION

Tuning Surface Reactivity and Electric Field Strength via Intermetallic Alloying

Ezra L. Clark[1], Rasmus Nielsen[1], Jakob Ejler Sørensen[1], Julius Lucas Needham[1], Brian Seger[1], Ib Chorkendorff[1]*

[1]SurfCat Section for Surface Physics and Catalysis, Department of Physics, Technical University of Denmark, 2800 Kgs Lyngby, Denmark

[*]Corresponding Author

Corresponding Author Contact: ibchork@fysik.dtu.dk

SUPPLEMENTARY INFORMATION

Table of Contents

SI-1 Intermetallic Alloy Selection…………………………………………………………………..3

SI-2 Experimental Methods…………………………..…………………………………………...7

SI-3 Energy Dispersive Spectroscopy of Intermetallic Thin Films ………….……………………………...9

SI-4 Grazing Incidence X-Ray Diffraction of Intermetallic Thin Films ………………..………………….10

SI-5 Intermetallic Thin Film Phase Identification……………………………………………….....11

SI-6 Scherrer and Williamson-Hall Analysis of XRD Data……………………………………………..12

SI-7 Extended X-Ray Photoelectron Spectroscopy of Ar Sputtered Intermetallic Thin Films ……….……13

SI-8 Low Energy He Ion Scattering of Ar Sputtered Intermetallic Thin Films …………………...………..14

SI-9 Validation of Work Function Measurements ………………………..……………………………..15

SI-10 Low Energy He Ion Scattering Depth Profile of Air Exposed PdGe …………………..…...………..16

SI-11 CO Reduction over Air Exposed and Segregated PdSn…………………………………………...17

SI-12 Deleterious Impact of Electrochemical Pretreatment on PdGe ……………………..…...………..18

SI-1 Intermetallic Alloy Selection

**Criteria 1**	Constituent B Metallic at Onset Potential of CO Reduction over Cu in 0.1 M KOH (-1.2 V vs SHE)
**Criteria 2**	Intermetallic Alloy Formation Energy Greater than CO Adsorption Energy to Cu(111) (-0.36 eV)
**Criteria 3**	AB Intermetallic Work Function Greater than Cu (4.48 eV)

**Constituent A Work Function**	**Constituent B**	**Constituent B Oxide Reduction Potential in 0.1 M KOH (V vs SHE)**	**Criteria 1**	**Intermetallic Alloy Formation Energy (eV)**	**Criteria 2**	**Constituent B Work Function (eV)**	**AB Intermetallic Work Function Assuming Linear Combination (eV)**	**Criteria 3**
Fe	Sc	-1.71						
4.70	Ti	-1.51						
	V	-1.49						
	Cr	-1.35						
	Y	-1.69						
	Zr	-1.63						
	Nb	-1.67						
	Mo	-0.90	PASS	0.00				
	Hf	-1.77						
	Ta	-1.72						
	W	-1.02	PASS	-0.02				
	Zn	-1.22						
	Ga	-1.35						
	Ge	-1.10	PASS	-0.11				
	As	-0.93	PASS	-0.27				
	Cd	-0.80	PASS	No Data				
	In	-1.06	PASS	No Data				
	Sn	-1.15	PASS	0.00				
	Sb	-0.92	PASS	-0.02				
	Te	-0.62	PASS	-0.18				
	Hg	0.06	PASS	No Data				
	Tl	-1.04	PASS	No Data				
	Pb	-1.14	PASS	No Data				
	Bi	-0.77	PASS	No Data				
Ru	Sc	-1.71						
4.71	Ti	-1.51						
	V	-1.49						
	Cr	-1.35						
	Y	-1.69						
	Zr	-1.63						
	Nb	-1.67						
	Mo	-0.90	PASS	-0.06				
	Hf	-1.77						
	Ta	-1.72						
	W	-1.02	PASS	-0.07				
	Zn	-1.22						
	Ga	-1.35						
	Ge	-1.10	PASS	-0.36				
	As	-0.93	PASS	-0.40	PASS	3.75	4.23	
	Cd	-0.80	PASS	No Data				
	In	-1.06	PASS	-0.14				
	Sn	-1.15	PASS	-0.22				
	Sb	-0.92	PASS	-0.25				
	Te	-0.62	PASS	-0.34				
	Hg	0.06	PASS	No Data				
	Tl	-1.04	PASS	No Data				
	Pb	-1.14	PASS	No Data				
	Bi	-0.77	PASS	No Data				
Os	Sc	-1.71						
5.93	Ti	-1.51						
	V	-1.49						
	Cr	-1.35						
	Y	-1.69						
	Zr	-1.63						
	Nb	-1.67						
	Mo	-0.90	PASS	0.00				
	Hf	-1.77						
	Ta	-1.72						
	W	-1.02	PASS	-0.06				
	Zn	-1.22						
	Ga	-1.35						
	Ge	-1.10	PASS	-0.09				
	As	-0.93	PASS	-0.22				
	Cd	-0.80	PASS	No Data				
	In	-1.06	PASS	No Data				
	Sn	-1.15	PASS	No Data				
	Sb	-0.92	PASS	-0.02				
	Te	-0.62	PASS	-0.07				
	Hg	0.06	PASS	No Data				
	Tl	-1.04	PASS	No Data				
	Pb	-1.14	PASS	No Data				
	Bi	-0.77	PASS	No Data				

Constituent A Work Function	Constituent B	Constituent B Oxide Reduction Potential in 0.1 M KOH (V vs SHE)	Criteria 1	Intermetallic Alloy Formation Energy (eV)	Criteria 2	Constituent B Work Function (eV)	AB Intermetallic Work Function Assuming Linear Combination (eV)	Criteria 3
Co	Sc	-1.71		[redacted]	[redacted]	[redacted]	[redacted]	[redacted]
5.00	Ti	-1.51		[redacted]	[redacted]	[redacted]	[redacted]	[redacted]
	V	-1.49		[redacted]	[redacted]	[redacted]	[redacted]	[redacted]
	Cr	-1.35		[redacted]	[redacted]	[redacted]	[redacted]	[redacted]
	Y	-1.69		[redacted]	[redacted]	[redacted]	[redacted]	[redacted]
	Zr	-1.63		[redacted]	[redacted]	[redacted]	[redacted]	[redacted]
	Nb	-1.67		[redacted]	[redacted]	[redacted]	[redacted]	[redacted]
	Mo	-0.90	PASS	-0.05		[redacted]	[redacted]	[redacted]
	Hf	-1.77		[redacted]	[redacted]	[redacted]	[redacted]	[redacted]
	Ta	-1.72		[redacted]	[redacted]	[redacted]	[redacted]	[redacted]
	W	-1.02	PASS	-0.08		[redacted]	[redacted]	[redacted]
	Zn	-1.22		[redacted]	[redacted]	[redacted]	[redacted]	[redacted]
	Ga	-1.35		[redacted]	[redacted]	[redacted]	[redacted]	[redacted]
	Ge	-1.10	PASS	-0.22		[redacted]	[redacted]	[redacted]
	As	-0.93	PASS	-0.33		[redacted]	[redacted]	[redacted]
	Cd	-0.80	PASS	No Data		[redacted]	[redacted]	[redacted]
	In	-1.06	PASS	-0.01		[redacted]	[redacted]	[redacted]
	Sn	-1.15	PASS	-0.12		[redacted]	[redacted]	[redacted]
	Sb	-0.92	PASS	-0.19		[redacted]	[redacted]	[redacted]
	Te	-0.62	PASS	-0.21		[redacted]	[redacted]	[redacted]
	Hg	0.06	PASS	No Data		[redacted]	[redacted]	[redacted]
	Tl	-1.04	PASS	No Data		[redacted]	[redacted]	[redacted]
	Pb	-1.14	PASS	No Data		[redacted]	[redacted]	[redacted]
	Bi	-0.77	PASS	No Data		[redacted]	[redacted]	[redacted]
Rh	Sc	-1.71		[redacted]	[redacted]	[redacted]	[redacted]	[redacted]
4.98	Ti	-1.51		[redacted]	[redacted]	[redacted]	[redacted]	[redacted]
	V	-1.49		[redacted]	[redacted]	[redacted]	[redacted]	[redacted]
	Cr	-1.35		[redacted]	[redacted]	[redacted]	[redacted]	[redacted]
	Y	-1.69		[redacted]	[redacted]	[redacted]	[redacted]	[redacted]
	Zr	-1.63		[redacted]	[redacted]	[redacted]	[redacted]	[redacted]
	Nb	-1.67		[redacted]	[redacted]	[redacted]	[redacted]	[redacted]
	Mo	-0.90	PASS	-0.24		[redacted]	[redacted]	[redacted]
	Hf	-1.77		[redacted]	[redacted]	[redacted]	[redacted]	[redacted]
	Ta	-1.72		[redacted]	[redacted]	[redacted]	[redacted]	[redacted]
	W	-1.02	PASS	-0.26		[redacted]	[redacted]	[redacted]
	Zn	-1.22		[redacted]	[redacted]	[redacted]	[redacted]	[redacted]
	Ga	-1.35		[redacted]	[redacted]	[redacted]	[redacted]	[redacted]
	Ge	-1.10	PASS	-0.56	PASS	5.00	4.99	PASS
	As	-0.93	PASS	-0.51	PASS	3.75	4.37	
	Cd	-0.80	PASS	No Data		[redacted]	[redacted]	[redacted]
	In	-1.06	PASS	-0.43	PASS	4.09	4.54	PASS
	Sn	-1.15	PASS	-0.48	PASS	4.42	4.70	PASS
	Sb	-0.92	PASS	-0.51	PASS	4.55	4.77	PASS
	Te	-0.62	PASS	-0.41	PASS	4.95	4.97	PASS
	Hg	0.06	PASS	-0.01		[redacted]	[redacted]	[redacted]
	Tl	-1.04	PASS	0.00		[redacted]	[redacted]	[redacted]
	Pb	-1.14	PASS	-0.09		[redacted]	[redacted]	[redacted]
	Bi	-0.77	PASS	-0.20		[redacted]	[redacted]	[redacted]
Ir	Sc	-1.71		[redacted]	[redacted]	[redacted]	[redacted]	[redacted]
5.42	Ti	-1.51		[redacted]	[redacted]	[redacted]	[redacted]	[redacted]
	V	-1.49		[redacted]	[redacted]	[redacted]	[redacted]	[redacted]
	Cr	-1.35		[redacted]	[redacted]	[redacted]	[redacted]	[redacted]
	Y	-1.69		[redacted]	[redacted]	[redacted]	[redacted]	[redacted]
	Zr	-1.63		[redacted]	[redacted]	[redacted]	[redacted]	[redacted]
	Nb	-1.67		[redacted]	[redacted]	[redacted]	[redacted]	[redacted]
	Mo	-0.90	PASS	-0.34		[redacted]	[redacted]	[redacted]
	Hf	-1.77		[redacted]	[redacted]	[redacted]	[redacted]	[redacted]
	Ta	-1.72		[redacted]	[redacted]	[redacted]	[redacted]	[redacted]
	W	-1.02	PASS	-0.36		[redacted]	[redacted]	[redacted]
	Zn	-1.22		[redacted]	[redacted]	[redacted]	[redacted]	[redacted]
	Ga	-1.35		[redacted]	[redacted]	[redacted]	[redacted]	[redacted]
	Ge	-1.10	PASS	-0.35		[redacted]	[redacted]	[redacted]
	As	-0.93	PASS	-0.48	PASS	3.75	4.59	PASS
	Cd	-0.80	PASS	-0.07		[redacted]	[redacted]	[redacted]
	In	-1.06	PASS	-0.16		[redacted]	[redacted]	[redacted]
	Sn	-1.15	PASS	-0.31		[redacted]	[redacted]	[redacted]
	Sb	-0.92	PASS	-0.38	PASS	4.55	4.99	PASS
	Te	-0.62	PASS	-0.30		[redacted]	[redacted]	[redacted]
	Hg	0.06	PASS	No Data		[redacted]	[redacted]	[redacted]
	Tl	-1.04	PASS	No Data		[redacted]	[redacted]	[redacted]
	Pb	-1.14	PASS	No Data		[redacted]	[redacted]	[redacted]
	Bi	-0.77	PASS	-0.03		[redacted]	[redacted]	[redacted]

SUPPLEMENTARY INFORMATION

Constituent A Work Function	Constituent B	Constituent B Oxide Reduction Potential in 0.1 M KOH (V vs SHE)	Criteria 1	Intermetallic Alloy Formation Energy (eV)	Criteria 2	Constituent B Work Function (eV)	AB Intermetallic Work Function Assuming Linear Combination (eV)	Criteria 3
Ni	Sc	-1.71						
5.01	Ti	-1.51						
	V	-1.49						
	Cr	-1.35						
	Y	-1.69						
	Zr	-1.63						
	Nb	-1.67						
	Mo	-0.90	PASS	-0.09				
	Hf	-1.77						
	Ta	-1.72						
	W	-1.02	PASS	-0.11				
	Zn	-1.22						
	Ga	-1.35						
	Ge	-1.10	PASS	-0.31				
	As	-0.93	PASS	-0.31				
	Cd	-0.80	PASS	-0.02				
	In	-1.06	PASS	-0.18				
	Sn	-1.15	PASS	-0.27				
	Sb	-0.92	PASS	-0.27				
	Te	-0.62	PASS	-0.22				
	Hg	0.06	PASS	No Data				
	Tl	-1.04	PASS	No Data				
	Pb	-1.14	PASS	No Data				
	Bi	-0.77	PASS	-0.03				
Pd	Sc	-1.71						
5.22	Ti	-1.51						
	V	-1.49						
	Cr	-1.35						
	Y	-1.69						
	Zr	-1.63						
	Nb	-1.67						
	Mo	-0.90	PASS	-0.10				
	Hf	-1.77						
	Ta	-1.72						
	W	-1.02	PASS	-0.04				
	Zn	-1.22						
	Ga	-1.35						
	Ge	-1.10	PASS	-0.54	PASS	5.00	5.11	PASS
	As	-0.93	PASS	-0.37	PASS	3.75	4.49	PASS
	Cd	-0.80	PASS	-0.42	PASS	4.08	4.65	PASS
	In	-1.06	PASS	-0.52	PASS	4.09	4.66	PASS
	Sn	-1.15	PASS	-0.55	PASS	4.42	4.82	PASS
	Sb	-0.92	PASS	-0.46	PASS	4.55	4.89	PASS
	Te	-0.62	PASS	-0.37	PASS	4.95	5.09	PASS
	Hg	0.06	PASS	-0.17				
	Tl	-1.04	PASS	-0.22				
	Pb	-1.14	PASS	-0.30				
	Bi	-0.77	PASS	-0.32				
Pt	Sc	-1.71						
5.64	Ti	-1.51						
	V	-1.49						
	Cr	-1.35						
	Y	-1.69						
	Zr	-1.63						
	Nb	-1.67						
	Mo	-0.90	PASS	-0.36				
	Hf	-1.77						
	Ta	-1.72						
	W	-1.02	PASS	-0.34				
	Zn	-1.22						
	Ga	-1.35						
	Ge	-1.10	PASS	-0.47	PASS	5.00	5.32	PASS
	As	-0.93	PASS	-0.52	PASS	3.75	4.70	PASS
	Cd	-0.80	PASS	-0.32				
	In	-1.06	PASS	-0.47	PASS	4.09	4.87	PASS
	Sn	-1.15	PASS	-0.58	PASS	4.42	5.03	PASS
	Sb	-0.92	PASS	-0.48	PASS	4.55	5.10	PASS
	Te	-0.62	PASS	-0.40	PASS	4.95	5.30	PASS
	Hg	0.06	PASS	-0.09				
	Tl	-1.04	PASS	-0.10				
	Pb	-1.14	PASS	-0.23				
	Bi	-0.77	PASS	-0.28				

Constituent A Work Function	Constituent B	Constituent B Oxide Reduction Potential in 0.1 M KOH (V vs SHE)	Criteria 1	Intermetallic Alloy Formation Energy (eV)	Criteria 2	Constituent B Work Function (eV)	AB Intermetallic Work Function Assuming Linear Combination (eV)	Criteria 3
Cu	Sc	-1.71		[redacted]	[redacted]	[redacted]	[redacted]	[redacted]
4.48	Ti	-1.51		[redacted]	[redacted]	[redacted]	[redacted]	[redacted]
	V	-1.49		[redacted]	[redacted]	[redacted]	[redacted]	[redacted]
	Cr	-1.35		[redacted]	[redacted]	[redacted]	[redacted]	[redacted]
	Y	-1.69		[redacted]	[redacted]	[redacted]	[redacted]	[redacted]
	Zr	-1.63		[redacted]	[redacted]	[redacted]	[redacted]	[redacted]
	Nb	-1.67		[redacted]	[redacted]	[redacted]	[redacted]	[redacted]
	Mo	-0.90	PASS	No Data		[redacted]	[redacted]	[redacted]
	Hf	-1.77		[redacted]	[redacted]	[redacted]	[redacted]	[redacted]
	Ta	-1.72		[redacted]	[redacted]	[redacted]	[redacted]	[redacted]
	W	-1.02	PASS	No Data		[redacted]	[redacted]	[redacted]
	Zn	-1.22		[redacted]	[redacted]	[redacted]	[redacted]	[redacted]
	Ga	-1.35		[redacted]	[redacted]	[redacted]	[redacted]	[redacted]
	Ge	-1.10	PASS	0.00		[redacted]	[redacted]	[redacted]
	As	-0.93	PASS	0.00		[redacted]	[redacted]	[redacted]
	Cd	-0.80	PASS	0.00		[redacted]	[redacted]	[redacted]
	In	-1.06	PASS	-0.01		[redacted]	[redacted]	[redacted]
	Sn	-1.15	PASS	-0.03		[redacted]	[redacted]	[redacted]
	Sb	-0.92	PASS	-0.04		[redacted]	[redacted]	[redacted]
	Te	-0.62	PASS	-0.07		[redacted]	[redacted]	[redacted]
	Hg	0.06	PASS	0.00		[redacted]	[redacted]	[redacted]
	Tl	-1.04	PASS	No Data		[redacted]	[redacted]	[redacted]
	Pb	-1.14	PASS	No Data		[redacted]	[redacted]	[redacted]
	Bi	-0.77	PASS	0.00		[redacted]	[redacted]	[redacted]
Ag	Sc	-1.71		[redacted]	[redacted]	[redacted]	[redacted]	[redacted]
4.52	Ti	-1.51		[redacted]	[redacted]	[redacted]	[redacted]	[redacted]
	V	-1.49		[redacted]	[redacted]	[redacted]	[redacted]	[redacted]
	Cr	-1.35		[redacted]	[redacted]	[redacted]	[redacted]	[redacted]
	Y	-1.69		[redacted]	[redacted]	[redacted]	[redacted]	[redacted]
	Zr	-1.63		[redacted]	[redacted]	[redacted]	[redacted]	[redacted]
	Nb	-1.67		[redacted]	[redacted]	[redacted]	[redacted]	[redacted]
	Mo	-0.90	PASS	No Data		[redacted]	[redacted]	[redacted]
	Hf	-1.77		[redacted]	[redacted]	[redacted]	[redacted]	[redacted]
	Ta	-1.72		[redacted]	[redacted]	[redacted]	[redacted]	[redacted]
	W	-1.02	PASS	0.00		[redacted]	[redacted]	[redacted]
	Zn	-1.22		[redacted]	[redacted]	[redacted]	[redacted]	[redacted]
	Ga	-1.35		[redacted]	[redacted]	[redacted]	[redacted]	[redacted]
	Ge	-1.10	PASS	0.00		[redacted]	[redacted]	[redacted]
	As	-0.93	PASS	0.00		[redacted]	[redacted]	[redacted]
	Cd	-0.80	PASS	-0.07		[redacted]	[redacted]	[redacted]
	In	-1.06	PASS	-0.03		[redacted]	[redacted]	[redacted]
	Sn	-1.15	PASS	-0.02		[redacted]	[redacted]	[redacted]
	Sb	-0.92	PASS	-0.02		[redacted]	[redacted]	[redacted]
	Te	-0.62	PASS	-0.06		[redacted]	[redacted]	[redacted]
	Hg	0.06	PASS	0.00		[redacted]	[redacted]	[redacted]
	Tl	-1.04	PASS	0.00		[redacted]	[redacted]	[redacted]
	Pb	-1.14	PASS	0.00		[redacted]	[redacted]	[redacted]
	Bi	-0.77	PASS	0.00		[redacted]	[redacted]	[redacted]
Au	Sc	-1.71		[redacted]	[redacted]	[redacted]	[redacted]	[redacted]
5.37	Ti	-1.51		[redacted]	[redacted]	[redacted]	[redacted]	[redacted]
	V	-1.49		[redacted]	[redacted]	[redacted]	[redacted]	[redacted]
	Cr	-1.35		[redacted]	[redacted]	[redacted]	[redacted]	[redacted]
	Y	-1.69		[redacted]	[redacted]	[redacted]	[redacted]	[redacted]
	Zr	-1.63		[redacted]	[redacted]	[redacted]	[redacted]	[redacted]
	Nb	-1.67		[redacted]	[redacted]	[redacted]	[redacted]	[redacted]
	Mo	-0.90	PASS	No Data		[redacted]	[redacted]	[redacted]
	Hf	-1.77		[redacted]	[redacted]	[redacted]	[redacted]	[redacted]
	Ta	-1.72		[redacted]	[redacted]	[redacted]	[redacted]	[redacted]
	W	-1.02	PASS	0.00		[redacted]	[redacted]	[redacted]
	Zn	-1.22		[redacted]	[redacted]	[redacted]	[redacted]	[redacted]
	Ga	-1.35		[redacted]	[redacted]	[redacted]	[redacted]	[redacted]
	Ge	-1.10	PASS	0.00		[redacted]	[redacted]	[redacted]
	As	-0.93	PASS	0.00		[redacted]	[redacted]	[redacted]
	Cd	-0.80	PASS	-0.18		[redacted]	[redacted]	[redacted]
	In	-1.06	PASS	-0.23		[redacted]	[redacted]	[redacted]
	Sn	-1.15	PASS	-0.19		[redacted]	[redacted]	[redacted]
	Sb	-0.92	PASS	-0.06		[redacted]	[redacted]	[redacted]
	Te	-0.62	PASS	-0.09		[redacted]	[redacted]	[redacted]
	Hg	0.06	PASS	0.00		[redacted]	[redacted]	[redacted]
	Tl	-1.04	PASS	-0.04		[redacted]	[redacted]	[redacted]
	Pb	-1.14	PASS	-0.08		[redacted]	[redacted]	[redacted]
	Bi	-0.77	PASS	-0.05		[redacted]	[redacted]	[redacted]

SI-2 Experimental Methods

Sputter Deposition

PdGe, Pd_xSn_{1-x}, and PdIn thin films were prepared by sputter deposition using an AJA ATC Orion magnetron sputtering system with 4 individual targets (Pd, Ge, Sn, and In). All sputtering was performed in Ar at a pressure of 2 mTorr. The thin films were deposited onto Si(100) wafers, which were cleaned by Ar sputtering for 5 min immediately before the deposition. Pd-based intermetallic thin films were deposited by co-sputtering Pd (99.95% Kurt J. Lesker) with either Ge (99.999% Kurt J. Lesker), Sn (99.994% Kurt J. Lesker), or In (99.998% Kurt J. Lesker) at a combined rate of roughly 1 Å/s to a thickness of 100 nm.

Energy Dispersive Spectroscopy

The bulk compositions of the thin films were measured using an FEI Quanta FEG 200 scanning electron microscope (SEM) equipped with a Oxford Instruments energy dispersive spectrometer (EDS). Elemental quantification was conducted by measuring the x-ray emission from the Pd, Sn, and In L edges and the Ge K edge upon excitation by an electron beam (10 kV). Each sample was analyzed at 10 distinct positions in order to assess the spatial uniformity of the measured bulk composition, which was <1 at. % for all thin films investigated.

X-Ray Diffraction

The crystal structures of the thin films were analyzed using a PAN-Analytical Empyrian x-ray diffractometer (XRD) using Cu Kα radiation (40 kV, 40 mA). The diffractometer was equipped with parallel beam optics during all grazing incidence XRD measurements, which were performed with an incident angle of 0.25°. Grazing incidence diffractograms were recorded using a step size of 0.01° and a dwell time of 5 s. Phase identification was performed using Malvern Panalytical's HighScore Plus software.

X-Ray Photoelectron Spectroscopy

The near-surface compositions of the thin films were measured using a Thermo Scientific ThetaProbe x-ray photoelectron spectrometer (XPS). XPS was performed using monochromatized Al Kα radiation. Core level spectra were recorded using a pass energy of 100 eV, a step size of 0.05 to 0.1 eV, a dwell time of 50 ms, and at least 20 integrated sweeps. Valence band spectra were recorded using a pass energy of 20 eV, a step size of 0.025 eV, a dwell time of 50 ms, and at least 50 integrated sweeps. Ar sputtering of the sample surface was performed using an Ar pressure of 2 x 10^{-7} mbar and a beam energy of 4 kV. The ion beam was rastered over a 4 x 4 cm^2 area during sputtering. Angle-resolved XPS (ARXPS) was performed by measuring photoelectrons ejected at angles of 20 to 80° relative to the surface normal using a 2D detector, which was divided into 32 separate signal channels. Thus, each signal output was derived from photoelectrons emitted from a 1.875° window. The energy scale of the observed spectra was calibrated by setting the C 1s binding energy to 284.8 eV. The Pd 3d, Sn 3d, and In 3d spectral features were fit to a single component, the Ge 3d spectral features were fit to either one or two individual

components (Ge^0 and Ge^{4+}) using the Thermo Avantage software. Elemental quantification was performed by integrating the signals from the Pd 3d, Pd 3p, Ge 3d, Sn 3d, and In 3d regions using a Shirley background and normalizing them by an internally calibrated relative sensitivity factor. Pd 3p was used for quantification in samples containing Ge due to the overlap of Pd 3d with a Ge auger electron. The penetration depth of the ARXPS measurements were calculated assuming the penetration depth of photoelectrons emitted normal to the surface was 10 nm, which is roughly equivalent to 3 mean free paths of the photoelectrons investigated herein.

Ultraviolet Photoelectron Spectroscopy

The work functions of the thin films were measured using a Thermofisher Scientific NEXSA XPS. Ar sputtering of the sample surface was performed using an Ar pressure of 2 x 10^{-7} mbar and a beam energy of 4 kV. The ion beam was rastered over a 3 x 3 cm^2 area during sputtering. The ultraviolet photoemission spectra are acquired using a helium discharge lamp, with principal photon energies at 21.2 eV (HeI) and 40.8 eV (HeII), while applying a negative bias of -5V to the sample to deconvolute the work function of the surface from that of the energy analyzer. Spectra were acquired using a pass energy of 2.0 eV, a step size of 0.01 eV, a dwell time of 50 ms, and at least 10 integrated sweeps. The energy scale of the observed spectra was calibrated using the inflection point of the Fermi edge.

Low Energy He Ion Scattering

The surface compositions of the thin films were measured using low energy He ion scattering (He-LEIS) performed in the same instrument above. He-LEIS was performed using a He pressure of 2 x 10^{-7} mbar and beam energy of 1 kV. The beam was not rastered during the measurement. Spectra were acquired using a retard ratio of 2.5, a step size of 1 eV, a dwell time of 50 ms, and a single sweep.

Temperature Programmed Desorption

The CO temperature programmed desorption measurements were carried out in a custom-built chamber. The sample is placed in a copper holder, which is cooled using liquid nitrogen. Isotopically labeled $C^{18}O$ is dosed until full coverage is achieved. The sample is heated using a tungsten alloy filament at a rate of 0.5 °K/s. The desorbed gas is measured using a quadrupole mass spectrometer (QMA 125, Pfeiffer Vacuum Technology AG).

SI-3 Energy Dispersive Spectroscopy of Intermetallic Thin Films

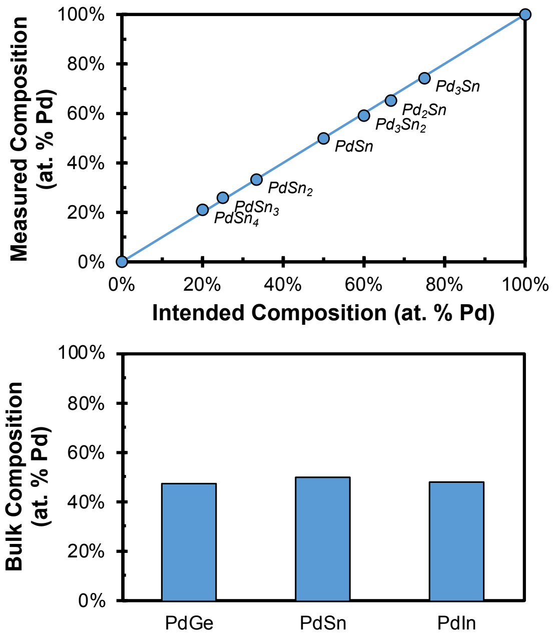

***Figure S1** – Bulk composition of Pd_xSn_{1-x}, PdGe, and PdIn intermetallic thin films, as measured by energy dispersive spectroscopy.*

SI-4 Grazing Incidence X-Ray Diffraction of Intermetallic Thin Films

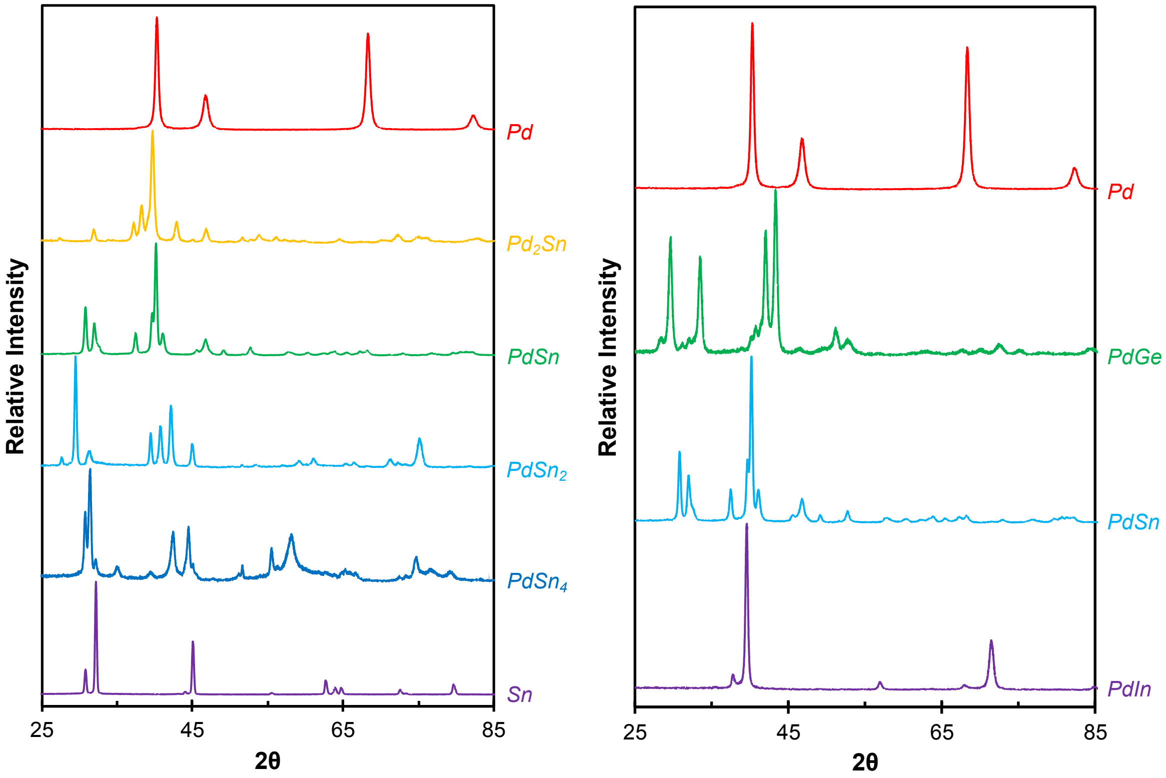

Figure S2 *– Grazing incidence x-ray diffractograms of Pd_xSn_{1-x}, PdGe, and PdIn intermetallic thin films.*

SI-5 Intermetallic Thin Film Phase Identification

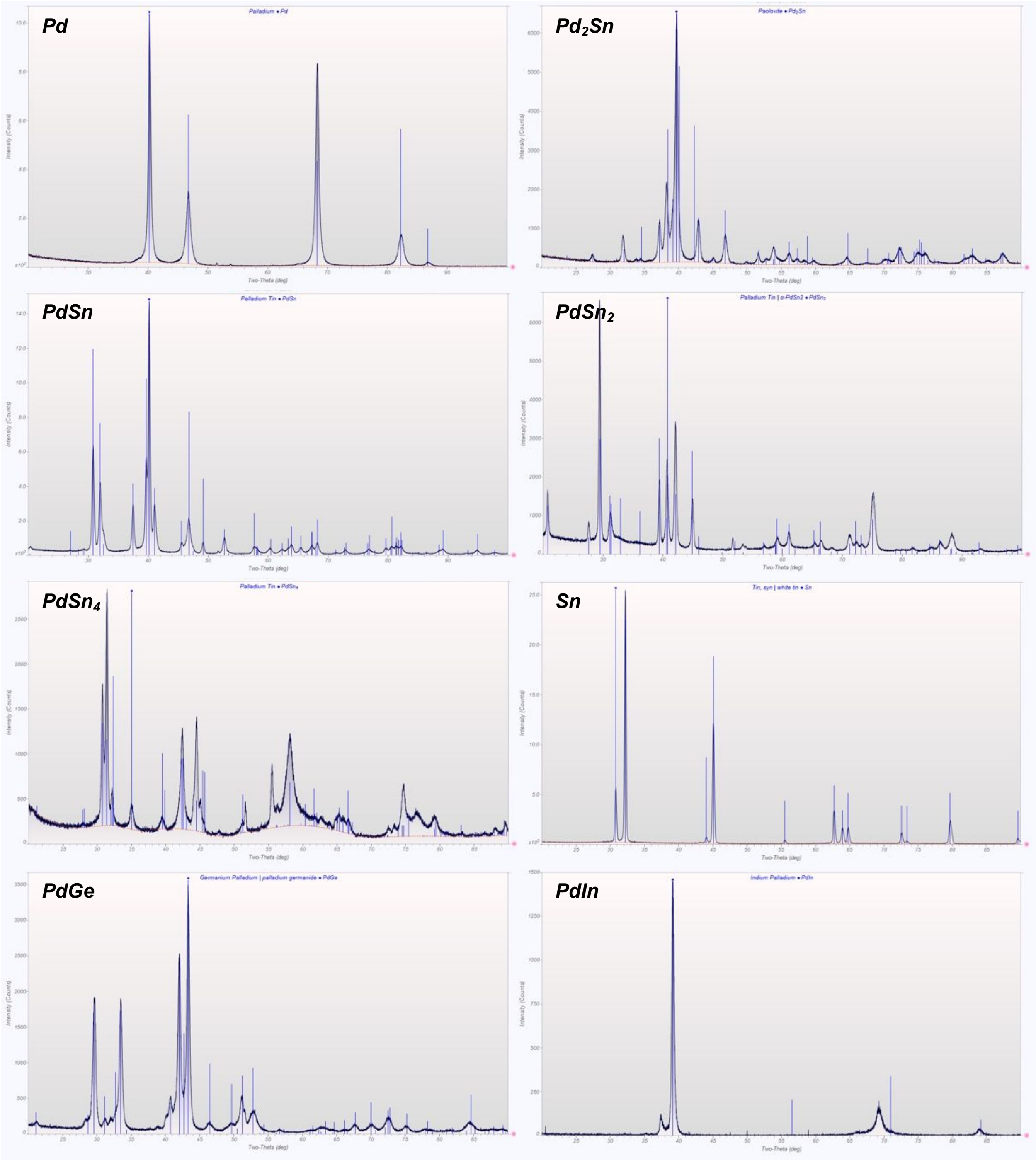

Figure S3 *– Comparison of the observed GIXRD patterns with reference data form the ICDD database.*

SI-6 Scherrer and Williamson-Hall Analysis of XRD Data

Table S1 *– Scherrer analysis of the XRD data.*

Phase	Average Particle Size (nm)
Pd	16.8
Pd_2Sn	19.5
PdSn	24.9
$PdSn_2$	26.2
$PdSn_4$	25.0
Sn	41.9
PdGe	18.7
PdIn	21.5

Table S2 *– Williamson-Hall analysis of the XRD data.*

Phase	Average Particle Size (nm)	Lattice Strain (%)
Pd	16.9	0.13%
Pd_2Sn	21.7	0.08%
PdSn	20.7	0.08%
$PdSn_2$	38.5	0.21%
$PdSn_4$	20.4	0.06%
Sn	32.2	0.02%
PdGe	25.2	0.29%
PdIn	26.7	0.24%

SI-7 Extended X-Ray Photoelectron Spectroscopy of Ar Sputtered Intermetallic Thin Films

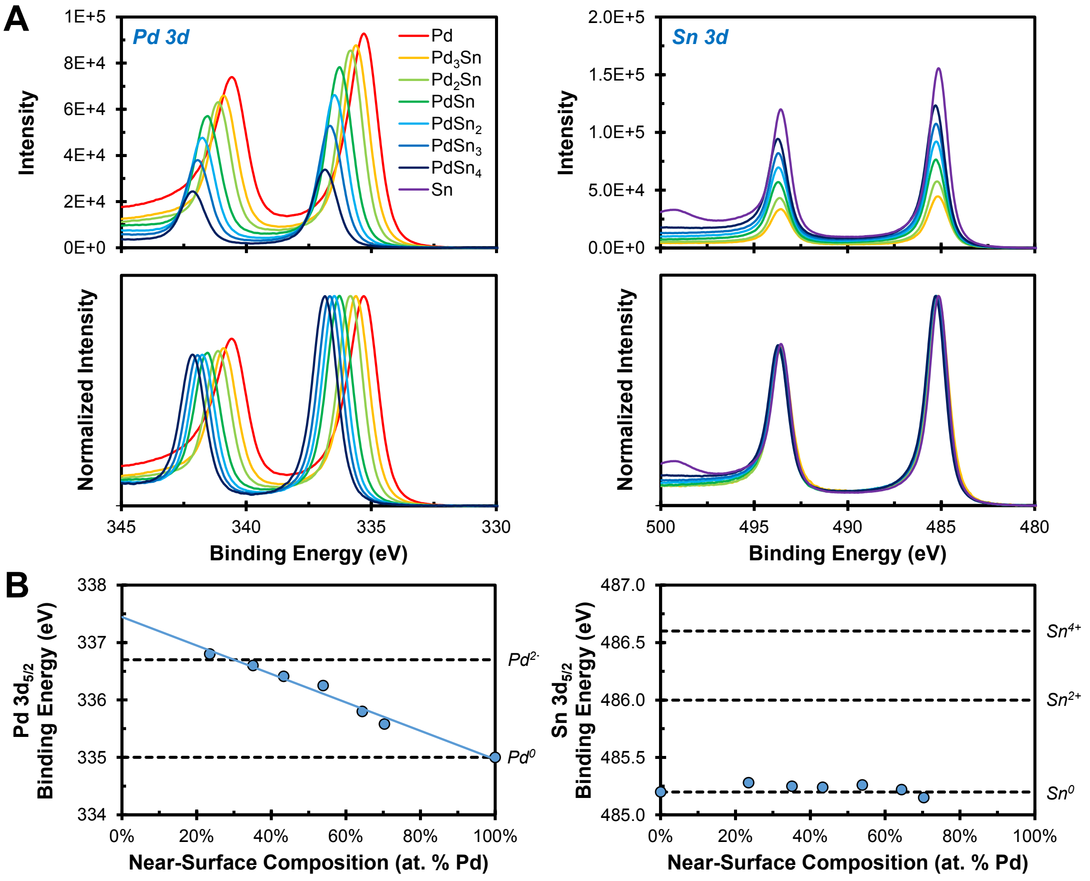

Figure S4 *– (A) X-ray photoelectron spectra of the Pd 3d and Sn 3d regions of Ar sputtered Pd_xSn_{1-x} intermetallic thin films. (B) Dependence of the Pd 3d and Sn 3d core level binding energies on near-surface composition.*

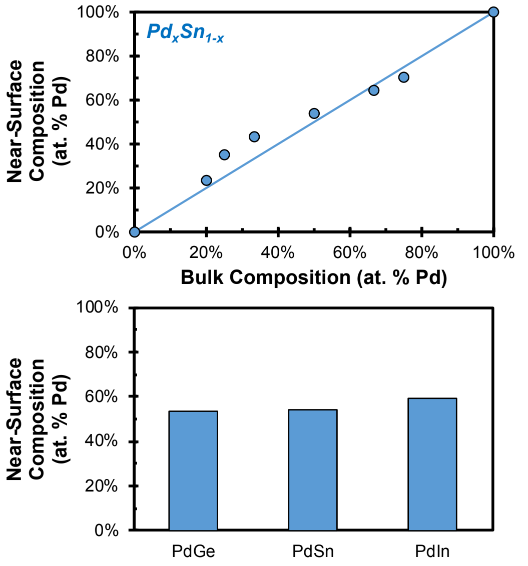

Figure S5 *– Near-surface composition of Ar sputtered Pd_xSn_{1-x}, PdGe, and PdIn intermetallic thin films, as measured by x-ray photoelectron spectroscopy.*

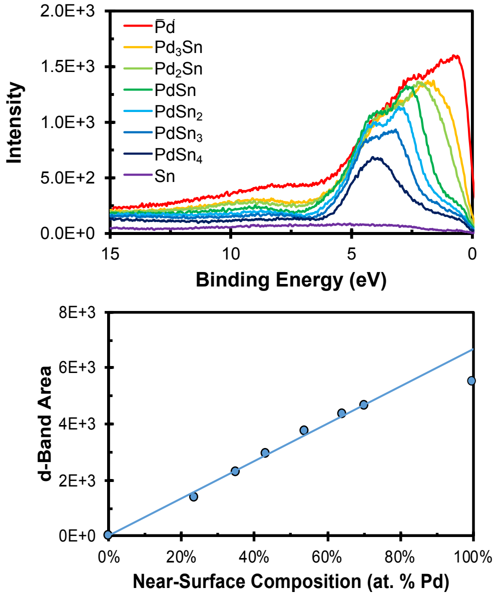

Figure S6 *– Raw XPS data of the Pd_xSn_{1-x} valence band region. Linear correlation between the integrated area of the d-band portion of the valence band and the near-surface Pd content of the Pd_xSn_{1-x} thin films.*

SI-8 Low Energy He Ion Scattering of Ar Sputtered Intermetallic Thin Films

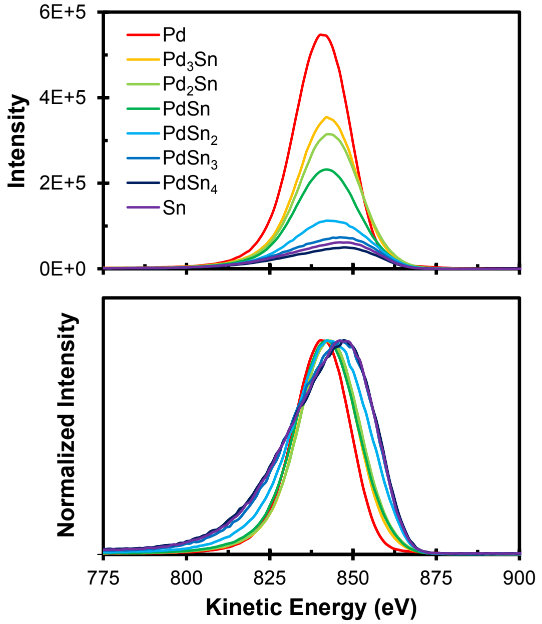

Figure S7 *– Low energy He ion scattering spectra of Ar sputtered Pd_xSn_{1-x} intermetallic thin films.*

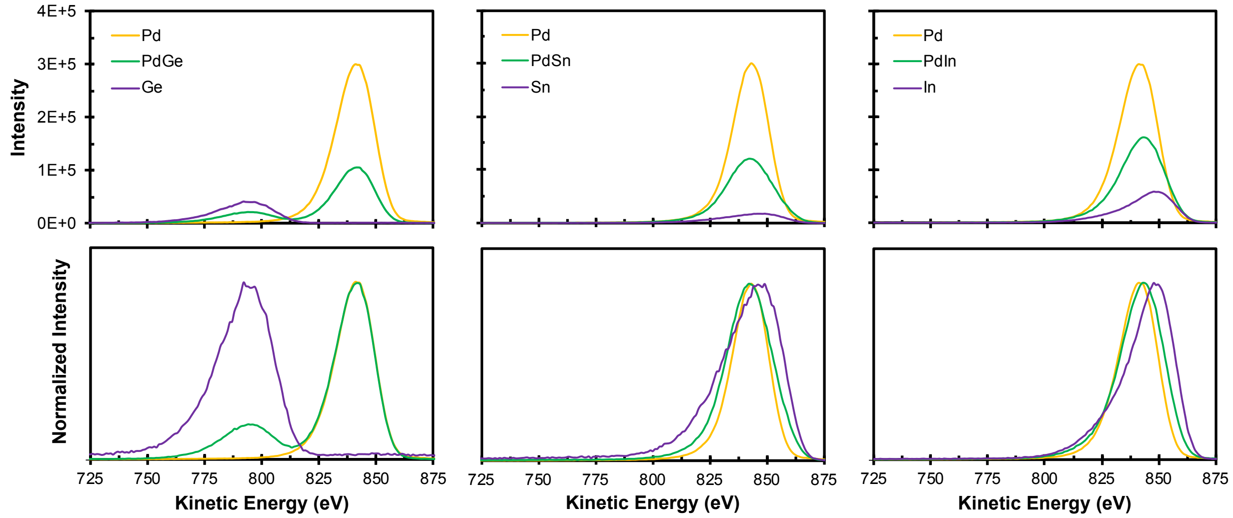

Figure S8 *– Low energy He ion scattering spectra of Ar sputtered PdGe, PdSn, and PdIn intermetallic thin films.*

SI-9 Validation of Work Function Measurements

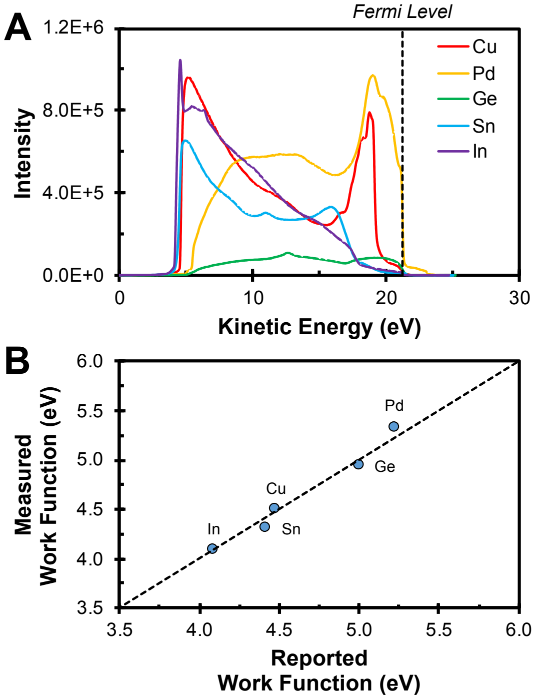

Figure S9 *– (A) Ultraviolet photoelectron spectra of Cu, Pd, Ge, Sn, and In. (B) Correlation between the work functions measured by ultraviolet photoelectron spectroscopy and those reported in the published literature.*

SI-10 Low Energy He Ion Scattering Depth Profile of Air Exposed PdGe

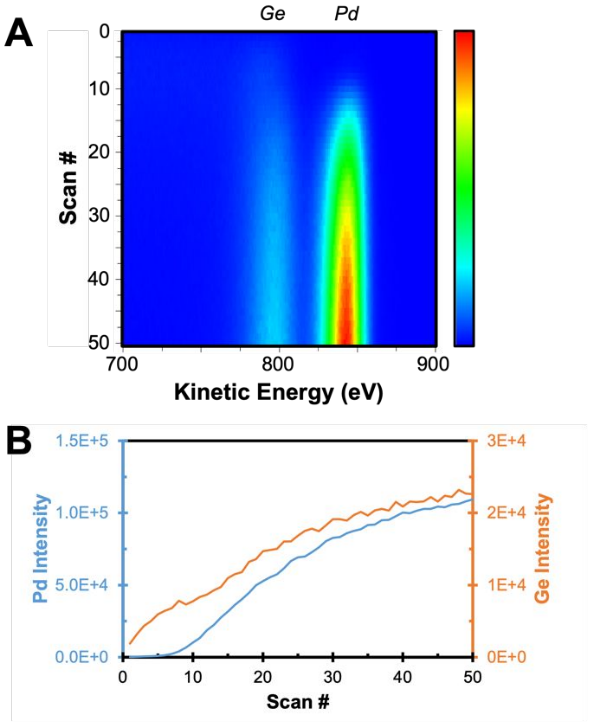

Figure S10 *– Low energy He ion scattering depth profile of air exposed PdGe. (A) Raw signals. (B) Pd and Ge intensity as a function of scan number.*

SI-11 CO Reduction over Air Exposed and Segregated PdSn

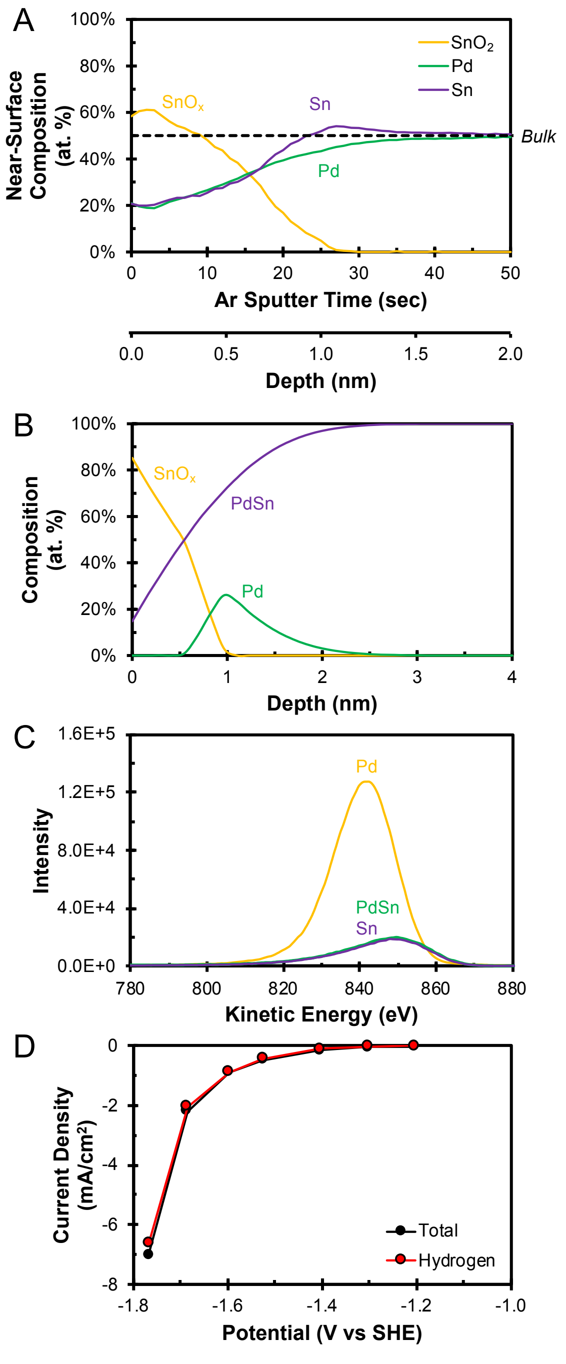

***Figure S11** – Characterization of air exposed PdSn: (A) Ar ion beam XPS depth profile. (B) Angle resolved XPS depth profile. (C) Low energy He ion scattering. Pd and Sn shown for comparison. (D) CO reduction chronoamperometry staircase.*

SI-12 Deleterious Impact of Electrochemical Pretreatment on PdGe

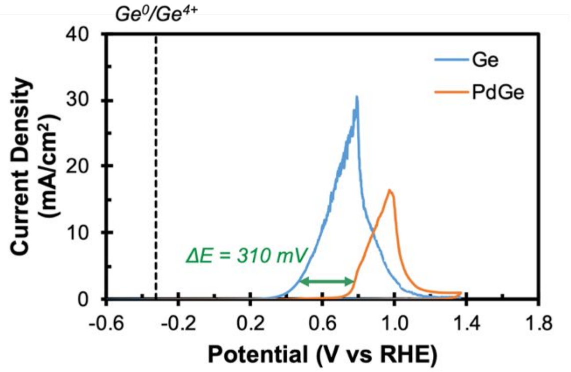

Figure S12 *– Cyclic voltammograms over Ge and PdGe in 0.1 M KOH at 50 mV/s exhibiting rapid Ge corrosion.*
